# A comparison of unheated loose housing with stables on the respiratory health of weaned-foals in cold winter conditions: an observational field-study

**DOI:** 10.1186/s13028-017-0339-3

**Published:** 2017-10-26

**Authors:** Reija Junkkari, Heli Simojoki, Minna-Liisa Heiskanen, Sinikka Pelkonen, Satu Sankari, Riitta-Mari Tulamo, Anna Mykkänen

**Affiliations:** 1 Department of Equine and Small Animal Medicine, University of Helsinki, Helsinki, 00014 Finland; 2 Department of Production Animal Medicine, University of Helsinki, Helsinki, 00014 Finland; 3 Equine Information Centre, Neulaniementie 5, Kuopio, 70210 Finland; 4 Research and Laboratory Department, Veterinary Bacteriology Research Unit, Finnish Food Safety Authority Evira, Neulaniementie 4, Kuopio, 70701 Finland

**Keywords:** Cold-winter loose-housing, Respiratory disease, Stables, Weaned-foals

## Abstract

**Background:**

Newly weaned horses in Finland are often moved to unheated loose housing systems in which the weanlings have free access to a paddock and a shelter. This practice is considered to be good for the development of young horses. The daily temperatures can stay below − 20 °C in Finland for several consecutive weeks during the winter season. However, the effect of unheated housing in a cold climatic environment on the respiratory health of weanlings under field conditions has not been studied before. This investigation was an observational field-study comprising 60 weanlings among 11 different voluntary participant rearing farms in Finland. Weanlings were either kept in unheated loose housing systems (n = 36) or in stables (n = 24) and were clinically examined on two separate occasions 58 days apart in cold winter conditions.

**Results:**

The odds of clinical respiratory disease were lower in the older foals (log_e_ days); OR = 0.009, P = 0.044). The plasma fibrinogen concentration was higher when the available space (m^2^/weanling) in the sleeping hall was smaller (P = 0.014) and it was lower when the sleeping hall was not insulated (P = 0.010). The plasma fibrinogen concentrations at the second examination were lower with a body condition score above 3 (P = 0.070). Standardbreds kept in loose housing systems had a lower body condition score than Finnhorses or Standardbreds kept in stables at both examinations (P = 0.026 and P = 0.007, respectively). Haemoglobin level was lower in weanlings in loose housing systems compared to their counterparts at the first examination (P = 0.037). Finnhorses had higher white blood cell count than Standardbreds at first (P = 0.002) and at the second examination (P = 0.001).

**Conclusions:**

Keeping weanling horses in cold loose housing systems does not seem to increase the occurrence of respiratory disease, but special attention should be focused on ventilation, air quality and feeding-practices. Our field study data suggest it might be advantageous to keep Standardbred foals born late in the season in a stable over the Finnish winter.

## Background

In a loose housing system, horses are kept in groups and where they can freely move between the paddock and the shelter. The loose housing practice is considered to be good for the development of young horses and foals because of the social contacts between individuals, fresh air and free movement [1–3]. The daily temperatures in winter in Finland and some other Nordic countries, fluctuate from − 14 to 0 °C and in certain areas of Finland they can fall below − 20 °C for several consecutive weeks [4]. After weaning at 5–6 months of age, the foals are moved to loose housing. This is done from October to December. We estimate that the proportion of foals kept in loose housing systems in Finland is around 30%. The cold housing environment increases the energy consumption of weanlings in early winter but not in late winter and weanlings gain weight at the expected rates when the energy consumed meets the increased demand caused by cold ambient temperature [5, 6].

Weaning [7, 8], transport [9, 10] and the inclusion into a new herd [11] causes substantial stress for foals. Stress, in turn, causes immunosuppression in young horses and increases the risk of respiratory disease [12]. Weanlings from several different farms are moved to central loose housing systems, and it is therefore highly likely that the whole group of weanlings in a centralised rearing facility will have encountered different pathogens earlier in life. Several viruses can cause respiratory infections in horses [13–18]. There is, however, increasing evidence that bacteria, especially *Streptococcus zooepidemicus*, can be a primary pathogen in respiratory tract infections in horses [19–21].

Certain laboratory tests can be useful in detecting equine respiratory disease or differentiating between non-infectious inflammation and viral or bacterial infection. A determination of blood white blood cell count (WBC) alone, may not be helpful in clinical cases because WBCs show quite substantial variation in healthy horses [22] and also between horses of different breeds [23]. On the other hand, low serum iron concentration is a good indicator for infection especially when combined with analysis for acute phase proteins such as serum amyloid A (SAA) and fibrinogen [24, 25]. The amount of SAA is negligible in a healthy horse but it increases rapidly in tissue trauma or in non-infectious or infectious inflammation cases and then it returns to baseline within 11–22 days [26, 27]. Plasma fibrinogen concentrations have a relatively wide normal range in the adult horse (2–4 g/L) [23]. The fibrinogen concentration increases over 24–72 h after the onset of inflammation and has a relatively long half-life of approximately 5 days [28]. Therefore, fibrinogen concentration is not as effective as SAA concentration is in detecting acute changes in disease progression but it does indicate a recent or ongoing infection. The plasma fibrinogen concentration of young horses seems to be higher than that of adult horses, and values can exceed 5 mg/L in healthy, 4–5-month-old horses [29, 30]. Both fibrinogen and SAA concentrations in foals with bacterial infections are higher than in foals with non-bacterial or uncertain diagnoses [31]. Reduced blood haemoglobin (Hb) concentration may indicate presence of a chronic infection [32]. Differentiating local infections from systemic infections in the horse is, however, challenging. This is also the case for respiratory diseases, which can be classified as occurring as a local event or as part of a systemic condition [33].

In addition to infectious agents, management and housing conditions influence the respiratory health of horses [34–36]. Pasture is considered to be a better environment than a stable [37–39]. The quality of air in a stable is affected by the bedding material, the feed and the ventilation [40–42]. The quality of the hay is the most important factor that affects the particulate concentration in air within the breathing zone of an adult horse, and placing hay on the floor is better than hanging the hay in a hay-net [40–43]. Horses need shelter during the winter months in northern latitudes. Horses in loose housing systems have access to a sleeping hall where the temperature is usually slightly higher than outside ambient temperatures. Little information is available on the effects of living in cold temperatures on respiratory disease in horses and in weanlings. Exercise in cold air can induce bronchoconstriction and inflammatory changes in the lower airways for 48 h after exercise [44, 45] and extended exposure to cold conditions has been reported to increase the risk of mucus accumulation in the trachea of adult horses [36]. However, there is no evidence that cold ambient temperature is associated with clinical respiratory disease in adult horses. Moreover, as far as the authors are aware the effect of cold housing environment on the respiratory health of weanlings has not been studied before.

The first aim of this study was to compare two management systems of cold loose housing and conventional stabling, in terms of the incidence of respiratory disease of weanling horses. We hypothesized that the incidence of respiratory disease would not differ between the management systems. The secondary aim was to determine whether certain environmental factors in loose housing systems or the breed of the horse affect the risk of clinical respiratory disease and any changes in inflammatory markers.

## Methods

This study was an observational field study of weanling horses in two different types of housing units that consists of two examinations taken 58 days apart.

### Horses, housing and management

A total of 60 newly weaned horses that were housed on 11 separate farms were examined (Table 1). The farms were selected by the owners’ willingness to participate and all weanling horses in these farms were included in the study. The weanlings were 139–232 days old (mean 187; SD 21) at the time of the first examination. Five of the farms had loose housing systems and six had conventional stables. The conditions in the loose housing farms (A–E) are presented in Table 2. Weanlings of the loose housing farms were kept in a group and were able to move freely to and from the shelter. The loose housing farms A and C had an entrance shelter, wind box and plastic sheets, farm B had an entrance shelter, farm D had plastic sheets suspended that covered the entrance and farm E had an entrance shelter and plastic sheets. An entrance shelter is a two-sided roofed shelter in front of the sleeping hall opening. A wind box is an entrance shelter that has plastic sheets over the outer door opening. Plastic sheets were 30–50 cm-wide strips that hung in the door opening to block the wind but allowed the weanlings to walk through. There were no adult horses in the same shelters or paddocks with the weanlings in the loose housing farms. There were only Finnhorses in the loose housing farm E. Other farms had both Standardbreds and Finnhorses. In the stables, weanlings were kept in individual stalls inside a barn and the stall doors opened to the barn aisle and adult horses were housed in the same corridor with the weanlings. Humidity and air particulate content were not measured. The weanlings spent the nights in the stables and were turned out into a paddock in a group during the daytime. All weanlings were fed haylage ad libitum outdoors and concentrate in the stable or the sleeping hall. The weanlings where tethered beside the feeding buckets during feeding. The haylage was analysed (Seilab Oy, Seinäjoki, Finland) and the quality was fairly similar among the farms. The farms were located in Eastern Finland, and the daily temperatures in the area ranged from − 17.7 to + 2.2 °C (mean − 3.5 °C) in December 2014 and − 21.3 to − 2.1 °C (mean − 7.1 °C) in January 2015 (Finnish Meteorological Institute).

**Table 1:** The breed, age and sex of the weanlings kept in different housing systems

Breed	n	Age/days	Loose housing	Stable	Colt	Filly
Standardbred	42	190 (148–232)	26	16	22	20
Finnhorse	18	179 (151–213)	10	8	9	9

The number, sex and mean (range) age at the first examination of the weanlings kept in loose housing systems and stables

**Table 2:** The conditions in loose housing systems

	A	B	C1	C2	D	E
Bedding	P	S	P + S	P + S	S	S
Insulation	x	x			x	x
Number of foals	3	10	10	4	4	5
Sleeping area/foal in m^2^	15	3	5	3	10	19

The structure and conditions in the five loose housing farms (A–E). On farm C there were two separate sleeping halls, C1 and C2

*P* peat, *S* straw, *P* *+* *S* straw on top of a peat base

### Clinical examination

The horses were examined on the farms at the beginning of the study in November–December 2014 and again 58 days later, in January 2015. Foals had been kept on the study farms for 2–50 (median 23; SD 16) days before the first visit. Special attention was paid to any signs of respiratory disease. The horses were considered to have a clinical respiratory disease based on the following criteria: temperature ≥ 38.3 °C and one or more of the following signs: cough, nasal/ocular discharge or increased respiratory sounds [14, 18]. The body condition (1: thin, 2: fair, 3: good, 4: fat, 5: very fat) was scored according to Carroll and Huntington [46].

### Blood analyses

Blood was drawn from the jugular vein into ethylenediaminetetraacetic acid (EDTA)-anticoagulated tubes for centrifugation and subsequent plasma analysis and also into plain tubes for coagulation and subsequent serum analysis during both farm visits. Samples were stored at room temperature for 6–8 h and then at + 4 °C until the samples were analysed within 48 h. Blood samples were taken from 60/60 weanlings during the first visit. Blood samples were obtained from 59/60 weanlings during the second visit, because one of the weanlings strongly resisted the sampling. Complete blood cell counts were analysed using an ADVIA 2120i Hematology System with multispecies software (Siemens Healthcare Diagnostics, Tarrytown, NY, USA) and using the cyanmethaemoglobin method for haemoglobin determination. Plasma fibrinogen was measured with the heat precipitation method [35]. An immunoturbidometric assay (Eiken SAA LZ, Eiken Chemical, Tokio, Japan) was carried out for serum amyloid A (SAA) and a colorimetric method for serum iron (Fe). The serum analyses were performed with an automatic clinical chemistry analyser (Konelab 30i, Thermo Fisher Scientific, Vantaa, Finland).

### Statistical analyses

After validation checks for data inconsistencies and missing entries, statistical analyses were carried out using Stata Intercooler version 11.0 (Stata Corporation, College Station, Texas, USA). Factors that affected clinical respiratory disease (0/1) at the first examination were analysed with a logistic mixed model. At the second examination, the proportion of clinically affected horses (6/60) was too low for statistical analysis. Factors that affected WBC, Hb, plasma fibrinogen and serum iron (Fe) at the first and second examinations were analysed by a linear mixed model. The plasma fibrinogen concentration was transformed using the natural logarithm in order to meet the assumption of a normal distribution.

Predictors in all models were the recumbency area available per weanling, body condition score (≤ 2.5, 3, ≥ 3.5), conditions of the stable (1–3), bedding (1–4), breed (Standardbred/Finnhorse), sex (colt/filly), type of stable (loose housing system/stall) and age of the foal (continuous predictor). Age was transformed to a natural logarithm to achieve a normal distribution. The recumbency area available per weanling was categorized into a dichotomous variable: below 8 m^2^ and over 8 m^2^. The conditions of the housing were categorized 1–3 as follows: 1: weanlings in a stable, 2: loose house stalls with insulation and 3: loose house stalls without insulation. The bedding types were 1: peat, 2: straw, 3: a mixture of straw and peat, 4: pelleted materials. The breed and sex of the foal were treated as confounders, so they were forced in all models. The age of the foal when it was moved to a loose housing system was available only for 36 foals, so this predictor could not be used in the models. Associations between all predictors in first and second examination and the outcomes were computed using simple logistic regression (clinical respiratory disease) or univariable regression analysis (fibrinogen, Hb, WBC, Fe). The predictors with association P ≤ 0.2 were included in the final models.

The full and final logistic model of clinical respiratory disease included age, conditions of the stable, bedding, sex and breed. The model fit was assessed using the assumptions of normality and homogeneity of variances, which were evaluated by checking the residuals. The final linear mixed model of fibrinogen at the first examination also included age, conditions of the stable, sex and breed. The final linear mixed model of fibrinogen at the second examination included the body condition score, conditions of the stable, sex and breed. Additionally, there was an interaction between the lying area and conditions of the stable, therefore the interaction term and lying area were also included in these models. The final linear mixed model of Hb at the first examination included type of stable, recumbency area available per horse, body condition score, sex and breed. The final linear mixed model of Hb at the second examination included age, sex and breed. The final linear mixed model of WBC at the first examination included age and breed. The final linear mixed model of WBC at the second examination included the breed. The final linear mixed model of Fe at the first examination included bedding, conditions of the stable, sex and breed. The final linear mixed model of Fe at the second examination included conditions of the stable, sex and breed. The farm was used as a random effect in all the models. Assumptions of the model were controlled using normality and scatter plots of the model residuals.

The association of the clinical respiratory disease with fibrinogen at the first examination was analysed by the Student’s t test. Test P ≤ 0.05 were considered to be statistically significant.

## Results

### Clinical respiratory disease

Assessment of the incidence of clinical respiratory disease was the principal objective. Clinical respiratory disease at the first examination was diagnosed in 28% (17/60) of the weanlings and 11 of these had a purulent nasal discharge. As much as 39% (14/36) of the loose housed weanlings compared 13% (3/24) stabled weanlings had a clinical respiratory disease at the first examination. The percentages at the second examination were 10% (6/60) for the whole study population, 11% (4/36) for loose housed weanlings and 8% (2/24) stabled. Three weanlings had purulent nasal discharge at the second examination. One weanling of the six with clinical respiratory disease had acute disease at the second examination and the other five had already manifested respiratory signs at the first examination. The greater age (log_e_ days) of the weanling decreased the odds ratio (OR) for having clinical respiratory disease (OR = 0.009, P = 0.044) at the first examination. As an interpretation, 2 weeks increase of age cause lower OR for clinical respiratory disease (OR = 0.56). The conditions in loose housing systems did not influence the occurrence of clinical disease. There was no statistical difference between the horses kept in stables or in loose housing systems. The breed or sex of the horse did not influence the occurrence of clinical disease.

### Plasma fibrinogen and haematology (secondary results)

**Fig. 1:**
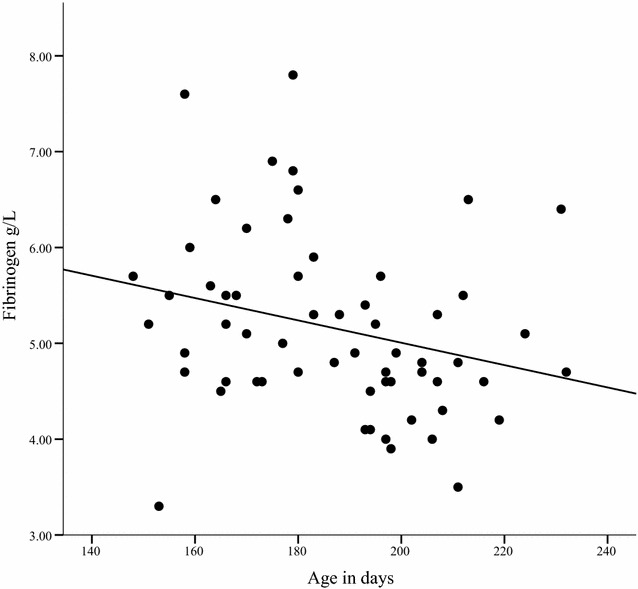
The plasma fibrinogen concentration (g/L) and age in days at the first examination of weanling horses, n = 60

**Fig. 2:**
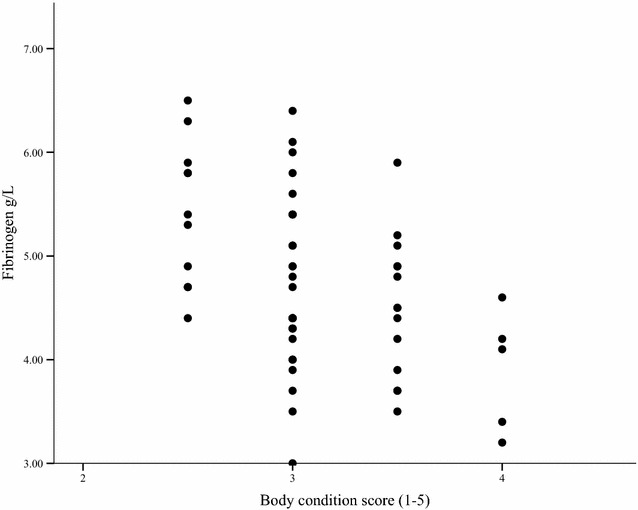
The plasma fibrinogen concentration (g/L) and body condition score at the second examination of weanling horses, n = 59

The plasma fibrinogen concentrations are presented in Tables 3 and 4. The fibrinogen concentration at the first examination was higher in younger horses (coeff = − 0.41, P = 0.033, Fig. 1) and in loose housing stalls where the lying area available per weanling was less than 8 m^2^ (coeff = 0.17, P = 0.014). Plasma fibrinogen concentrations were lower if the sleeping hall was not insulated (coeff = − 0.22, P = 0.01) compared to fibrinogen concentrations of stabled weanlings. The weanlings with clinical respiratory disease had a higher plasma fibrinogen concentration at the first examination (P < 0.001). The fibrinogen concentration at the second examination was higher in weanlings that had a body condition score under 3 compared to weanlings that had a body condition score of 3 (coeff = − 0.60, P = 0.022) or above 3 (coeff = − 0.75, P = 0.015) (Fig. 2). Neither the sex nor the breed of the foal had an effect on the fibrinogen concentration at either examination.

**Table 3:** The mean haematological values of Standardbred weanlings

	1st visit stable	1st visit Lh	2nd visit stable	2nd visit Lh
Serum SAA (mg/L)	0.9 [± 0.5]	1.2 [± 1.6]	0.7 [± 0.4]	4.8 [± 17.8]
Plasma fibrinogen (g/L)	5.0 [± 0.6]	5.4 [± 1.1]	4.6 [± 0.6]	5.1 [± 0.7]
WBC (10^9^/L)^a^	12.0 [± 2.4]	12.2 [± 2.3]	10.4 [± 1.8]	11.7 [± 2.6]
Fe (µmol/L)	24 [± 9.4]	29 [± 7.9]	23 [± 4.7]	27 [± 7.0]
Hb (g/L)	141 [± 11.4]	145 [± 11.5]	134 [± 8.9]	130 [0 ± 10.8]

The mean haematological values [± SD] of Standardbred weanlings kept in loose housing (Lh), n = 26, and in stables, n = 16

*SAA* serum amyloid A, *WBC* white blood cell count, *Hb* haemoglobin

^a^Signifies being statistically different from the Finnhorse

**Table 4:** The mean haematological values of Finnhorse weanlings

	1st visit stable	1st visit Lh	2nd visit stable	2nd visit Lh
Serum SAA (mg/L)	0.4 [± 0.5]	12 [± 24.2]	1.3 [± 2.9]	0.8 [± 0.5]
Plasma fibrinogen (g/L)	4.8 [± 0.9]	5.0 [± 0.9]	4.2 [± 0.9]	4.2 [± 1.0]
WBC (10^9^/L)^a^	14.8 [± 3.9]	14.2 [± 2.0]	14.3 [± 4.0]	15.1 [± 2.8]
Fe (µmol/L)	23 [± 6.8]	24 [± 9.7]	24 [± 7.7]	21 [± 7.5]
Hb (g/L)	132 [± 16.7]	123 [± 10.8]	120 [± 9.5]	117 [± 5.1]

The mean haematological values [± SD] in serum and plasma of Finnhorse weanlings kept in loose housing (Lh), n = 9, and in stables, n = 8

*SAA* serum amyloid A, *WBC* white blood cell count, *Hb* haemoglobin

^a^Signifies being statistically different from the Standardbred

At both examination times, Finnhorse weanlings had a higher WBC count than Standardbreds; coeff = 2.22, P = 0.002 and coeff = 3.61, P < 0.001, respectively. By contrast, Hb was lower in Finnhorse than in Standarsbreds at the first (coeff = − 12.60, P < 0.001) and second (coeff = − 12.66, P < 0.001) examination. The horses kept in loose housing systems had lower Hb at the first examination (coeff = 8.41, P = 0.037) but not at the second examination.

Fe did not differ between the two breeds (Tables 3, 4). There was no association between a high WBC count and clinical respiratory disease. There was no statistically significant difference in Fe or SAA values between healthy and clinically affected horses. There was no difference in Fe values between loose housing system and stable but the values at the first examination were lower in those farms where mixture of peat and straw was used as bedding material (coeff = 9.26, P = 0.001) compared to peat bedding. Serum SAA exceeded the laboratory reference value in two horses at the first examination and one at the second examination.

### Body condition score

The body condition scores at both examinations are presented in Table 5. The Standardbred weanlings housed in a stable had a higher body condition score than those kept in loose housing systems at both examinations (P = 0.026 and P = 0.007, respectively). Mean (± SD) body condition score in Standardbreds in loose housing systems at the first examination was 2.7 (± 0.5) and 3.1 (± 0.4) for the stable weanlings. The mean scores at the second examination were 2.8 (± 0.3) and 3.2 (± 0.2), respectively. The mean body condition score in the Finnhorse weanlings in loose housing systems at the first examination was 3.7 (± 0.5) and 3.3 (± 0.7) those housed in the stables. The mean scores at the second examination were 3.6 (± 0.5) and 3.3 (± 0.4), respectively.

**Table 5:** Body condition scores

	≤ 2.5	3	≥ 3.5
Standardbred Lh (n = 26)	14^a^/10^a^	10/14	2/2
Standardbred stable (n = 16)	2^a^/0^a^	11/11	3/5
Finnhorse Lh (n = 10)	0/1	2/0	8/9
Finnhorse stable (n = 8)	2/0	2/4	4/4

The number of horses categorized by the body condition score (1: thin, 2: fair, 3: good, 4: fat, 5: very fat)

*Lh* loose housing, *n* number of horses in each group, *x/y* number of horses in each category at the first/second examination

^a^Signifies being statistically different between stable and loose housing systems

## Discussion

To our knowledge, this is the first study on respiratory disease in weanlings living in a cold climate. We found that, a young age of the weanling was the most important factor associated with the occurrence of clinical respiratory disease. There was no difference in the occurrence of clinical respiratory disease in weanlings housed in stables compared to loose housing systems. Heleski et al. [2] reported that regardless of the method of weaning, the blood cortisol levels of foals peak at 4 weeks post-weaning. The time from weaning to the first examination was not recorded in this present study. Therefore, it cannot be concluded whether this stress peak affected the risk of clinical disease. No differences were detected in relation to the breed or sex of the weanling in terms of clinical disease.

High particulate and endotoxin concentrations in the breathing zone of a horse are known to induce inflammatory changes in the airways [43, 47]. Bedding [40, 48, 49], ventilation [50] and the quality and feeding method of the hay [40, 43] affect the air quality in animal shelters. In the loose housing farms the haylage was provided outside and in the stable farms also inside in the night time. The quality of the bedding was hence emphasized in the loose housing farms compared to stables when it comes to air particulate content inside the shelter because it was only potentially dusty material in sleeping halls. The humidity or air particulate concentration were not measured but air quality was estimated to be good in all stables and sleeping halls. We found that the bedding material or other differences in conditions did not affect the risk of clinical disease in cold loose housing. However, serum iron was found to be significantly lower when the bedding used in the sleeping hall was a mixture of peat and straw. This bedding combination was used in only one of the loose housing farms so it is possible that low iron concentration was associated with other factors on this farm and not the type of the bedding used.

The WBC count was higher and Hb values were lower in Finnhorses compared with Standardbreds at both examinations. This was an unexpected finding because the WBC reference values are lower for adult cold-blooded horses [23]. During the blood sampling, there was no difference in behaviour between the breeds that could explain the result. Blood haemoglobin concentration was lower in horses in loose housing systems at the first examination. Low haemoglobin concentration can be a sign of chronic infection or inadequate nutrition. The mean body condition score of the Standardbred weanlings kept in loose housing systems was significantly lower than those kept in a stable and Finnhorses in either management system (Table 2). There were both Standardbred and Finnhorse weanlings together in all but one loose housing farm that had only Finnhorses and the quality of the haylage did not differ among the farms. The weanlings on the loose housing farms were tied to the wall beside the feeding bucket when eating the concentrate feed. Standardbred weanlings hence had equal opportunities as the Finnhorse weanlings to access the concentrate feed and the haylage. Jørgensen et al. [51] reported that the adult Warmblood horses chose heated shelter more often than the Coldblood horses when weather was rainy and windy [51]. Since haylage was only available outside the shelter, it is possible that the Standardbred weanlings might have spent more time in the shelter and therefore ate less than their Finnhorse counterparts. This was not however the case in a study by Autio et al. [6], who showed that there was no difference between Standardbred and Finnhorse weanlings in respect of the time spent in the shelter/paddock. Although the energy consumed was not recorded in this study, it is also plausible that Standardbred weanlings might require more energy in cold temperatures than Finnhorses and feeding should be planned according to the breed.

Plasma fibrinogen concentrations were higher in cramped sleeping halls and when the sleeping hall was insulated. The air quality was not recorded in this study. In an earlier study made in the previous year (Airaksinen et al., not published), air humidity in these same, insulated sleeping halls was recorded to be higher than outside and in one sleeping hall ammonia was also detected. In that same study, the quality of the straw used in the loose housing farms varied and there were also mouldy straw bales among the ones used. During our visits, the air quality was good but the ambient temperature, quality of bedding material and the handling of the bedding in the shelter can vary on a daily basis and there might have been some prevailing air quality problems that were not detectable during the short time of our two visits. It is possible that the weanlings ranking lower in the hierarchy were unable to utilize fully the more cramped sleeping halls, as is also the situation among adult horses when the recumbency area is too small in relation to the group size [52]. Haylage was provided ad libitum from round bales on every loose housing farm involved in this study. Weanlings on the stable farms were fed haylage on the ground. Eating hay from round bales has been associated with an increased number of neutrophils in the airways of fully grown adult horses [36]. Environmental factors could also partly explain why inflammatory markers did not correlate with the clinical symptoms at the second examination. A study on calves showed that a poor group housing environment might enhance the risk of respiratory disease without changes in the blood leukocyte response [53].

The plasma fibrinogen concentration had a negative correlation with age. This finding is in accordance with the study by Harvey et al. [29] and Santos et al. [30], both of which reported the plasma fibrinogen concentration to be highest at the age of 3–5 months after which it decreases gradually until the age of 6–12 months. The mean age of the weanlings in the present study was 6.2 months at the time of the first examination. The plasma fibrinogen concentration in healthy Thoroughbreds in the same age group as used in this study was reported to be less than 4.5 g/L [29, 30]. At the first examination, the mean fibrinogen concentration in this study in clinically healthy horses (4.9 g/L) was higher than in previous reports but the range was wide (3.5–7.8 g/L). The fibrinogen concentration was associated with clinical disease at the first (P = 0.004) but not at the second examination. It seems that the younger weanlings especially, caught respiratory infections when moved to new herd but they recovered during the study period. The weanlings that had a high fibrinogen concentration at the first examination were however likely to have a higher fibrinogen concentration and a low body condition score at the end of the study period.

The definition of clinical disease was determined in the present study by combining the definitions from two previous studies on equine respiratory health [14, 18]. Due to the long distances to the farms, the weanlings could only be examined twice and plasma fibrinogen was chosen as a marker of infection because of its long half-life [54]. The plasma fibrinogen concentration is a significant but unspecific predictor of inflammatory disease and is often combined with serum iron and SAA concentration in clinical practice [24, 25, 33]. In this study neither the serum SAA nor hypoferremia was associated with clinical respiratory disease. This was unexpected because serum SAA and iron have been shown to be more sensitive and rapid inflammatory markers than the WBC count or plasma fibrinogen concentration [25, 26, 31, 33, 55]. The sample storage and analysis method used in the present study should not have influenced the serum SAA concentration [52, 53]. It is possible that there was no specific infectious agent that caused the respiratory symptoms. One weakness of our study is that mild clinical symptoms might not have been noticed if they had appeared occasionally. Respiratory disease can also be local and hence not induce the systemic signs of inflammation in the blood. The time the weanlings had spent in a new herd and housing facility before the first examination was not constant in this field study, which could have affected the results. However, the time the weanling had spent on the farm was indeed evaluated but had missing values and therefore was not included. The number of horses in the study was quite small (n = 60). For these reasons, the power of the study is low, which might have impacted on the results and thus some important factors could be underestimated. The region of Eastern Finland was chosen because of its low winter temperatures, which was an essential predisposing factor in this study, but this choice also limited the geographical area, which in turn limited the actual number of foal rearing farms that could be contacted. Moreover, only a proportion of the foal rearing farms in this limited geographical area volunteered to be participants. Nevertheless, this study was conducted under field conditions, which are the same as those encountered by veterinary practitioners in this region. A full evaluation of the influence of certain environmental factors requires a study design with a larger population of horses in more precisely defined conditions is required.

It would also be worthwhile to make a nationwide survey for weanling owners to evaluate the occurrence of respiratory disease in different management systems because veterinarians only detect cases that require medical attention.

## Conclusions

The occurrence of respiratory disease and fibrinogen concentrations were higher as the weanlings were younger in this study. Contrary to our expectations, fibrinogen concentrations were higher in animals living in the loose housing systems that had an insulated sleeping hall compared to those that live in systems with noninsulated sleeping hall or in the stable. Standardbred weanlings kept in loose housing had lower body condition scores than Finnhorses or those kept in a stable. Standardbreds in loose housing systems also had lower blood haemoglobin concentration at the first examination. According to our results Standardbred weanlings seem to have a higher energy demand in a cold environment compared to that of their Finnhorse counterparts. Keeping weanlings in cold loose housing systems does not seem to increase the occurrence of respiratory disease, but special attention should be focused on ventilation, air quality and feeding practices. Our data suggest it might be advantageous to keep Standardbred foals born late in the season in a stable over the winter. Further studies with more weanlings and farms are warranted to define the optimal loose housing conditions for weanlings under cold climate conditions.

## Abbreviations

EDTA: ethylenediaminetetraacetic acid
Fe: iron
Hb: haemoglobin
Lh: loose housing
P: peat
P + S: a mixture of peat and straw
S: straw
SAA: serum amyloid A
WBC: white blood cell count

## References

[1] Zeep K, Schintzer U (1997). Housing and training of horses according to their species-specific behavior. Livest Prod Sci..

[2] Heleski CR, Shelle AC, Nielsen BD, Zanella AJ (2002). Influence of housing on weanling horse behavior and subsequent welfare. Appl Anim Behav Sci.

[3] Søndergaard E, Ladewig J (2004). Group housing exerts a positive effect on the behaviour of young horses during training. Appl Anim Behav Sci.

[4] Finnish Meteorologigal institute, Swedish Meteorologigal and hydrologigal institute.

[5] Cymbaluk NF, Christison GI (1998). Effects of diet and climate on growing horses. J Anim Sci.

[6] Autio E, Heiskanen ML (2005). Foal behaviour in loose housing/paddock environment during winter. Appl Anim Behav Sci.

[7] Erber R, Wulf M, Rose-Meierhoefer S, Becker-Birck M, Moestl E, Aurich J (2012). Behavioral and physiological responses of young horses to different weaning protocols: a pilot study. Stress..

[8] Henry S, Zanella AJ, Sankey C, Richard-Yris MA, Marko A, Hausberger M (2012). Adults may be used to alleviate weaning stress in domestic foals (*Equus caballus*). Physiol Behav.

[9] Smith BL, Jones JH, Hornof WJ, Miles JA, Longworth KE, Willits NH (1996). Effects of road transport on indices of stress in horses. Equine Vet J.

[10] Schmidt A, Hödl S, Möstl E, Aurich J, Müller J, Aurich C (2010). Cortisol release, heart rate, and heart rate variability in transport-naive horses during repeated road transport. Domest Anim Endocrinol.

[11] Alexander SL, Irvine CH (1998). The effect of social stress on adrenal axis activity in horses: the importance of monitoring corticosteroid-binding globulin capacity. J Endocrinol.

[12] Seung-Ho R, Koo HC, Park YK, Jung KW, Davis WC, Park YH (2009). Etiologic and immunologic characteristics of thoroughbred horses with bacterial infectious upper respiratory disease at the Seoul race park. J Microbiol Biotechnol.

[13] Dunowska M, Wilks CR, Studdert MJ, Meers J (2002). Equine respiratory viruses in foals in New Zealand. N Z Vet J..

[14] Newton JR, Wood JLN, Chanter N (2003). A case control study of factors and infections associated with clinically apparent respiratory disease in UK Thoroughbred racehorses. Prev Vet Med..

[15] Vairo S, Vandekerckhove A, Steukers L, Glorieux S, Van den Broeck W, Nauwynck H (2012). Clinical and virological outcome of an infection with the Belgian equine arteritis virus strain 08P178. Vet Microbiol.

[16] Horsington J, Hartley CA, Gilkerson JR (2015). Seroprevalence study of Equine rhinitis B virus (ERBV) in Australian weanling horses using serotype-specific ERBV enzyme-linked immunosorbent assays. J Vet Diagn Invest.

[17] Diaz-Mendez A, Viel L, Hewson J, Doig P, Carman S, Chambers T, Tiwari A, Dewey C (2010). Surveillance of equine respiratory viruses in Ontario. Can J Vet Res.

[18] Back H, Ullman K, Berntson TL, Riihimäki M, Penell J, Ståhl K (2015). Viral load of equine herpesviruses 2 and 5 in nasal swabs of actively racing standardbred trotters: temporal relationship of shedding to clinical findings and poor performance. Vet Microbiol.

[19] Erol E, Locke SJ, Donahoe JK, Mackin MA, Carter CN (2012). Beta-hemolytic *Streptococcus* spp. from horses: a retrospective study (2000–2010). J Vet Diagn Invest.

[20] Lindahl SB, Aspán A, Båverud V, Paillot R, Pringle J, Rash NL (2013). Outbreak of upper respiratory disease in horses caused by *Streptococcus equi* subsp. *zooepidemicus* ST-24. Vet Microbiol.

[21] Velineni S, Desoutter D, Perchec AM, Timoney JF (2014). Characterization of a mucoid clone of *Streptococcus zooepidemicus* from an epizootic of equine respiratory disease in New Caledonia. Vet J..

[22] Anhold H, Candon R, Chan DS, Amos W (2014). A comparison of elevated blood parameter values in a population of thoroughbred racehorses. J Equine Vet Sci..

[23] Jain CN. The horse: normal hematology with comments on response to disease. In: Schalm’s veterinary hematology 4th edition. Philadelphia: Lea & Febiger; 1986. p. 154–5.

[24] Borges AS, Divers TJ, Stokol T, Mohammed OH (2007). Serum iron and plasma fibrinogen concentrations as indicators of systemic inflammatory diseases in horses. J Vet Intern Med.

[25] Corradini I, Armengou L, Viu J, Rodríguez-Pozo ML, Cesarini C, Jose-Cunilleras E (2014). Parallel testing of plasma iron and fibrinogen concentrations to detect systemic inflammation in hospitalized horses. J Vet Emerg Crit Care..

[26] Hultén C, Sandgren B, Skiöldebrand E, Klingeborn B, Marhaug G, Forsberg M (1999). The acute phase protein serum amyloid A (SAA) as an inflammatory marker in equine influenza virus infection. Acta Vet Scand.

[27] Belgrave RL, Dickey MM, Arheart KL, Cray C (2013). Assessment of serum amyloid A testing of horses and its clinical application in a specialized equine practice. J Am Vet Med Assoc.

[28] Coyne CP, Hornof WJ, Kelly AB, O’Brien TR, DeNardo SJ (1985). Rapid extraction, radioiodination, and in vivo catabolism of 125I-labeled fibrinogen in the horse. Am J Vet Res.

[29] Harvey JW, Asquith RL, McNulty PK, Kivipelto J, Bauer JE (1984). Haematology of foals up to one year old. Equine Vet J.

[30] Santos FCC, Feijó LS, Kasinger S, Junior FF, Curcio BR, Nogueira CEW (2014). Hematologic values of Thoroughbred foals from birth to six months of age. Ciencia Anim Bras..

[31] Hultén C, Demmers S (2002). Serum amyloid A (SAA) as an aid in the management of infectious disease in the foal: comparison with total leucocyte count, neutrophil count and fibrinogen. Equine Vet J.

[32] Reed SM, Bayly WM, Sellon DC. Disorders of specific body systems. In: Equine internal medicine 3 rd edition. St. Louis: Saunders; 2010. p. 736-40.

[33] Hooijberg EH, van den Hoven R, Tichy A, Schwendenwein I (2014). Diagnostic and predictive capability of routine laboratory tests for the diagnosis and staging of equine inflammatory disease. J Vet Intern Med.

[34] Burrell MH, Wood JL, Whitwell KE, Chanter N, Mackintosh ME, Mumford JA (1996). Respiratory disease in thoroughbred horses in training: the relationships between disease and viruses, bacteria and environment. Vet Rec..

[35] Ivester KM, Smith K, Moore GE, Zimmerman NJ, Couëtilt LL (2012). Variability in particulate concentrations in a horse training barn over time. Equine Vet J Suppl..

[36] Robinson NE, Karmaus W, Holcombe SJ, Carr EA, Derksen FJ (2006). Airway inflammation in Michigan pleasure horses: prevalence and risk factors. Equine Vet J.

[37] Holcombe SJ, Jackson C, Gerber V, Jefcoat A, Berney C, Eberhardt S (2001). Stabling is associated with airway inflammation in young Arabian horses. Equine Vet J.

[38] Whittaker AG, Love S, Parkin TD, Duz M, Hughes KJ (2009). Stabling causes a significant increase in the pH of the equine airway. Equine Vet J.

[39] Berndt A, Derksen FJ, Edward Robinson N (2010). Endotoxin concentrations within the breathing zone of horses are higher in stables than on pasture. Vet J..

[40] Vandenput S, Istasse L, Nicks B, Lekeux P (1997). Airborne dust and aeroallergen concentrations in different sources of feed and bedding for horses. Vet Q..

[41] Clements JM, Pirie RS (2007). Respirable dust concentrations in equine stables. Part 1: validation of equipment and effect of various management systems. Res Vet Sci.

[42] Hessel EF, Garlipp F, Van den Weghe HFA (2009). Generation of airborne particles from horse feeds depending on type and processing. J Equine Vet Sci..

[43] Ivester KM, Couëtil LL, Moore GE, Zimmerman NJ, Raskin RE (2014). Environmental exposures and airway inflammation in young thoroughbred horses. J Vet Intern Med.

[44] Davis MS, Royer CM, McKenzie EC, Williamson KK, Payton M, Marlin D (2006). Cold air-induced late-phase bronchoconstriction in horses. Equine Vet J Suppl..

[45] Davis MS, Williams CC, Meinkoth JH, Malayer JR, Royer CM, Williamson KK, McKenzie EC (2007). Influx of neutrophils and persistence of cytokine expression in airways of horses after performing exercise while breathing cold air. Am J Vet Res.

[46] Carroll CL, Huntington PJ (1998). Body condition scoring and weight estimation of horses. Equine Vet J.

[47] Millerick-May ML, Karmaus W, Derksen FJ, Berthold B, Holcombe SJ, Robinson NE (2013). Local airborne particulate concentration is associated with visible tracheal mucus in thoroughbred racehorses. Equine Vet J.

[48] Airaksinen S, Heiskanen ML, Heinonen-Tanski H, Laitinen J, Laitinen S, Linnainmaa M (2005). Variety in dustiness and hygiene quality of peat bedding. Ann Agric Environ Med..

[49] Saastamoinen M, Särkijärvi S, Hyyppä S (2015). Reducing respiratory health risks to horses and workers: a comparison of two stall bedding materials. Animals..

[50] Wålinder R, Riihimäki M, Bohlin S, Hogstedt C, Nordquist T, Raine A (2011). Installation of mechanical ventilation in a horse stable: effects on air quality and human and equine airways. Environ Health Prev Med.

[51] Jørgensen GH, Aanensen L, Mejdell CM, Bøe KE (2016). Preference for shelter and additional heat in horses exposed to Nordic winter conditions. Equine Vet J.

[52] Baumgartner M, Zeitler-Feicht MH, Wohr AC, Wohling H, Erhard MH (2015). Lying behaviour of group-housed horses in different designed areas with rubber mats, shavings and sand bedding. Pferdeheilkunde..

[53] Cobb CJ, Obeidat BS, Sellers MD, Pepper-Yowell AR, Ballou MA (2014). Group housing of Holstein calves in a poor indoor environment increases respiratory disease but does not influence performance or leukocyte responses. J Dairy Sci.

[54] Crisman MV, Scarrat WK, Zimmerman KL (2008). Blood proteins and inflammation in the horse. Vet Clin N Am Equine Pract..

[55] Hultén C, Grönlund U, Hirvonen J, Tulamo RM, Suominen MM, Marhaug G (2002). Dynamics in serum of the inflammatory markers serum amyloid A (SAA), haptoglobin, fibrinogen and alpha2-globulins during induced noninfectious arthritis in the horse. Equine Vet J.

